# 

**Published:** 1887-04

**Authors:** 


					﻿IN MEMORIAM.
Dr Wm. Jefferson Burt,...................Austin,	Texas.
Died July io, 1886.
Dr. Hays White Moore,...............Ft. Worth, Texas.
Died Sept. 26, 1886.
Dr. Joseph S. Willis,..................... Waco,	Texas.
Died July 6, 1887.
Dr. Thomas M. Stone,.....................Jasper,	Texas.
Died Sept. 14, 1886.
Dr. Albert Welch,....................... Ferris,	Texas.
Died Sept. 26, 1886.
Dr. J. T. Meek,........................Ennis, Texas.
Died Nov. 11, 1886.
Dr. James Haley,.......................Moffatt, Texas.
Died Feb. 20, 1887.
Induce every M. D. to subscribe for Daniel’s Texas Medical
Journal. Only $2 a year, in advance.
				

